# Supplementary material for: Iterative improvement in the automatic modular design of robot swarms
Source: PeerJ Comput Sci. 2020 Dec 7;6:e322. doi: 10.7717/peerj-cs.322 (PMC7924708; doi:10.7717/peerj-cs.322)
Supplement: Supplemental Information 3 [file peerj-cs-06-322-s003.zip › argos3/doc/api/standalone/a00638_source.html]

ARGoS: plugins/simulator/visualizations/qt-opengl/qtopengl\_camera.h Source File


- Main Page
- Related Pages
- Namespaces
- Classes
- Files

- File List
- File Members

# plugins/simulator/visualizations/qt-opengl/qtopengl\_camera.h

Go to the documentation of this file.

```
00001 
00007 #ifndef QTOPENGL_CAMERA_H
00008 #define QTOPENGL_CAMERA_H
00009 
00010 namespace argos {
00011    class CQTOpenGLCamera;
00012 }
00013 
00014 class QPoint;
00015 
00016 #include <QObject>
00017 #include <argos3/core/utility/datatypes/datatypes.h>
00018 #include <argos3/core/utility/math/ray3.h>
00019 #include <argos3/core/utility/configuration/argos_configuration.h>
00020 
00021 #ifdef __APPLE__
00022 #include <gl.h>
00023 #include <glu.h>
00024 #else
00025 #include <GL/gl.h>
00026 #include <GL/glu.h>
00027 #endif
00028 
00029 namespace argos {
00030 
00031    class CQTOpenGLCamera : public QObject {
00032 
00033       Q_OBJECT
00034 
00035    public:
00036 
00037       struct SSettings {
00039          CVector3 Position;
00041          CVector3 Left;
00043          CVector3 Up;
00045          CVector3 Forward;
00047          CVector3 Target;
00049          Real LensFocalLength;
00051          CDegrees YFieldOfView;
00053          Real MotionSensitivity;
00055          Real RotationSensitivity;
00056 
00057          SSettings() :
00058             Position(-2.0f, 0.0f, 2.0f),
00059             Left(CVector3::Y),
00060             Up(CVector3(1.0f, 0.0f, 1.0f).Normalize()),
00061             Forward(CVector3(1.0f, 0.0f, -1.0f).Normalize()),
00062             Target(),
00063             LensFocalLength(0.02f),
00064             MotionSensitivity(0.005),
00065             RotationSensitivity(0.01) {
00066             CalculateYFieldOfView();
00067          }
00068 
00070          void Init(TConfigurationNode& t_tree);
00072          void RotateUpDown(const CRadians& c_angle);
00074          void RotateLeftRight(const CRadians& c_angle);
00076          void RotateLeftRight2(const CRadians& c_angle);
00078          void Translate(const CVector3& c_delta);
00080          void Do();
00082          void CalculateYFieldOfView();
00084          void CalculateSensitivity();
00085       };
00086 
00087    public:
00088 
00089       CQTOpenGLCamera();
00090       ~CQTOpenGLCamera();
00091 
00092       void Init(TConfigurationNode& t_tree);
00093 
00094       inline void Look() {
00095          m_sSettings[m_unActiveSettings].Do();
00096       }
00097 
00098       inline const CVector3& GetPosition() const {
00099          return m_sSettings[m_unActiveSettings].Position;
00100       }
00101 
00102       inline const CVector3& GetTarget() const {
00103          return m_sSettings[m_unActiveSettings].Target;
00104       }
00105 
00106       inline Real GetLensFocalLength() const {
00107          return m_sSettings[m_unActiveSettings].LensFocalLength;
00108       }
00109 
00110       void Rotate(const QPoint& c_delta);
00111 
00112       void Move(SInt32 n_forwards_backwards,
00113                 SInt32 n_sideways,
00114                 SInt32 n_up_down);
00115 
00116       inline SSettings& GetActiveSettings() {
00117          return m_sSettings[m_unActiveSettings];
00118       }
00119 
00120       inline const SSettings& GetActiveSettings() const {
00121          return m_sSettings[m_unActiveSettings];
00122       }
00123 
00124       inline void SetActiveSettings(UInt32 un_settings) {
00125          m_unActiveSettings = un_settings;
00126       }
00127 
00128       inline SSettings& GetSetting(UInt32 n_index) {
00129          return m_sSettings[n_index];
00130       }
00131 
00132    private:
00133 
00134       UInt32 m_unActiveSettings;
00135       SSettings m_sSettings[12];
00136 
00137    };
00138 
00139 }
00140 
00141 #endif
00142
```

---

Generated on 10 Jul 2018 for ARGoS by 
 1.6.1
